# Sports-related leisure behavior in Alpine regions during the COVID-19 pandemic—A cross-sectional study in Austria, Germany and Italy

**DOI:** 10.3389/fpubh.2023.1136191

**Published:** 2023-03-09

**Authors:** Anna-Maria Kogler, Stefanie Elisabeth Schöttl

**Affiliations:** Department of Sport Science, University of Innsbruck, Innsbruck, Austria

**Keywords:** COVID-19, pandemic, leisure behavior, sports events, travel behavior, Alpine region

## Abstract

**Introduction:**

As a planetary health challenge, COVID-19 forced governments around the world to take action to prevent the most severe consequences resulting from the spread of the virus. These measures ranged from stay-at-home orders to limitations on indoor and outdoor activities, travel restrictions and the cancelation of sports events, all of which affected people's leisure activities and daily lives. Therefore, the aim of this study is to examine changes in sports-related leisure behavior in terms of attending major sports events, following major sports events via media, travel intentions and the use of new sports offerings. Furthermore, we aimed to identify variables associated with changed sports-related leisure behavior during the pandemic.

**Methods:**

A cross-sectional online survey (*n* = 1,809) was conducted from December 2020 to January 2021 in the Alpine regions of Austria, Germany and Italy. Sports-related leisure behavior was investigated for changes during the pandemic compared to the time before the coronavirus outbreak and for differences between the three countries.

**Results:**

Results showed that the self-reported importance of attending major sports events decreased significantly in the Alpine regions of all three countries during COVID-19. Prevailing restrictions affected vacation planning for over 80% of participants. A majority of approximately three quarters of respondents stated they had spent their holidays at home during the travel restrictions. Sports facilities and opportunities played an important role in the choice of vacation destination for more than half of participants. A binary logistic regression showed significant correlations between vacation planning during COVID-19 and the variables gender, income, quality of life and mental health. 31.9% of all respondents tried new sports offerings during extended restrictions, with a large proportion (72.4%) using apps, online tools or courses. Furthermore, approximately 30% of respondents increased their e-sports consumption.

**Discussion:**

The results showed that sports-related leisure behavior in Alpine regions changed in the course of the COVID-19 pandemic. In the future, policymakers as well as sports and leisure providers should react to these behavioral changes and adapt their portfolio and services to meet consumers92 demands.

## 1. Introduction

The concept of leisure has been debated in many forums, from sociology to psychology and has various meanings. Traditionally, leisure is defined in terms of work, or rather the absence of work, and can be described as the time an individual has left after subtracting the time spent on work, sleep and other basic needs (1). According to Venkatesh, travel is a profound part of leisure behavior. An attempt to identify the factors that influence leisure and leisure travel behavior yields the following determinants: personality, motivation, attitude and situational and environmental factors (1). By contrast, leisure constraints hinder recreational activities (2) and can be categorized as intrapersonal (e.g., negative emotions), interpersonal (e.g., no company for leisure activities) and structural constraints (e.g., insufficient time, financial burden) (2, 3). However, these three constraints could be related to COVID-19 (3) and could possibly cause a deeper distinction or shift of each type of constraint in relation to engaging in leisure activities. Behavior itself can be influenced by a number of factors, including personal economic wellbeing and disposable income, changes in costs, perceived health risks, and changes in consumption capacity as a result of pandemic restrictions (4).

Ongoing studies continue to examine the impact of the COVID-19 pandemic on recreational behavior, leisure, travel and wellbeing, and quality of life, respectively (3, 5). As a result of social distancing measures, government-mandated restrictions on group leisure activities as well as on indoor and outdoor sports, people spent their leisure time during the pandemic, among other things, by engaging in home-based physical activities (6). The availability of home equipment was found to be an important predictor of physical activity in the home environment during strict COVID-19 lockdown measures (7). Changes in the type of physical activity were also observed during the initial lockdown measures with endurance, muscular strength and multimodal exercises being the most popular at this time (8). In addition, an increase in sports participation among less active groups of Tyrolean adults was observed during the period when people were ordered to stay at home (9). Also, a trend toward outdoor sports, especially ski touring on groomed slopes was observed in the Alpine region of Austria, and was probably accelerated by the restrictions resulting from the COVID-19 pandemic (e.g., closure of ski facilities) and the factor of time (e.g., short-time working hours), leading to an increased engagement in ski touring (10). Given that the World Health Organization (WHO) had recommended online gaming as a way of providing social interaction in times of severe measures (11), it is likely that the amount of screen time and participation in gaming and e-sports experienced a marked increase (12–14). In addition, Schöttl et al. showed, that people in the Alpine regions of Austria, Germany and Italy substituted their original physical activity behavior by taking online courses or exercising *via* apps, buying new sports equipment, practicing new types of sports, or doing home workouts (15).

As a further measure to prevent an increase in the number of infections, countries around the world canceled or postponed major events such as conferences, concerts, festivals and sports events, and banned gatherings of more than a certain number of people (16). Major sports leagues in Europe, North America, and other countries ended their seasons early and big sports events were canceled or postponed, including the 2020 Summer Olympics and the UEFA EURO 2020, which took place in the same countries in 2021 (16–18). The suspension of professional sports was followed by events without spectators, which were referred to as “ghost games” (19). Taking into account the measures in place at the time, sports organizers tried to ensure the resumption of games while encouraging people back to the events. However, following the introduction of spectator restrictions, the stadium experience changed profoundly (17, 20, 21). Fans were only allowed to gather in the spectators' stands in accordance with the social distancing regulations. Joyful dances, which express the spectators' passion for athletes and teams, could not be held in densely packed stands. It is clear, however, that being in a stadium gives fans a strong sense of social identity (21). Given that sports events are an important part of society (22), it is crucial to find out what factors influenced spectators' intentions to attend sports events during the COVID-19 crisis, and how flexible consumers who are accustomed to attending or participating in major sports events are when the circumstances of participation change radically. It also raises the question of how this potential change in behavior or shift in preferences might affect future product development by sports activity providers. COVID-19 could lead to a transformation of the sports industry in several ways (23), and with all of these changes, sports providers will need to reinvent themselves to deliver quality services to existing customers and to develop new perspectives to reach new customers, such as spectators, who will remain non-attendees of sports events even when constraints are lifted. The spectator's focus has likely shifted to a wider range of sports content due to rapid digitalization and increased opportunities for fan engagement (23). Technologies became particularly involved in fan engagement when people were able to support their teams online. Fans are also likely to engage with sports in other ways, such as through extended reality technologies. As new opportunities in online sports streaming emerge, home viewing has the potential to grow (23). A new perspective on the sports movie industry and off-field sports content could be gained (23), with the COVID-19 experience serving as a learning resource for sports event providers.

The impact of the COVID-19 pandemic on (travel) risk perception and travel behavior has been the subject of recent studies (24, 25). In this context, Neuburger and Egger showed a significant increase in COVID-19 risk perception, travel risk perception and the willingness to change or cancel travel plans in Germany, Austria and Switzerland, 2 weeks before and immediately after COVID-19 was declared a pandemic (24). Furthermore, the perception of COVID-19 as a risk associated with travel led to a planned change in a number of typical travel behaviors. The study by Bratic et al. shows that based on beliefs around COVID-19, people planned to change their choice of destination, accommodation, and travel activities, and even decided not to travel or to travel for a shorter period of time (25).

According to our literature research, it is apparent that indoor and outdoor leisure activities changed profoundly in the midst of the COVID-19 pandemic (3). To our knowledge, however, research that deals with cross-national changes in sports-related leisure behavior amid the COVID-19 pandemic is scant. Thus, the present study aims to examine how individuals from the Alpine regions in Austria, Germany and Italy negotiated leisure constraints and why leisure activities changed during the COVID-19 pandemic. Given its commonalities across countries, the Alpine region lends itself to comparison. The Alpine region is characterized by common regional features and complex topography such as mountains, forests and rich natural landscapes (26). The topographical and climatic conditions provide locals with countless opportunities for outdoor activities and recreation in nature (27, 28). Our study covered the following five Alpine regions: Tyrol (Austria), Vorarlberg (Austria), Upper Bavaria (Germany), South Tyrol (Italy) and Trentino (Italy). These regions are characterized by a well-organized sports infrastructure on a voluntary, private and public level (29). Comparing the three countries in the context of COVID-19 restrictions, Italy was hit the hardest in the first phase of the pandemic. Due to the extent of the virus' spread, more stringent measures were implemented in Italy than in Austria and Germany (30). The Oxford COVID-19 Index of Government Response Stringency can be used to objectively compare the extensive government response to the pandemic in the three countries (31). At the beginning of the first lockdown, Italy scored highest (93.52), followed by Austria (81.48) and Germany (76.85) on a scale from 0 to 100 (=strictest) (31). During the curfews, governments in all three countries restricted social contact and forced non-essential businesses, bars, restaurants, public parks as well as sports and leisure facilities to close. Travel in general, including air travel, was also suspended and hotels and accommodation establishments were forced to close. Events were also prohibited and home office advisories were issued. Only essential services (such as supermarkets, pharmacies, and hospitals) remained open, which went hand in hand with social distancing rules in public places and the mandatory wearing of masks indoors and on public transport (24, 32).

Therefore, the aim of this study is to investigate changes in leisure behavior and sports-related activities due to COVID-19 restrictions in the Alpine regions. More precisely, the objectives of this study are to (1) examine changes in sports-related leisure behavior in terms of attending major sports events, following major sports events *via* media, travel intentions and the use of alternative and new sports offerings; (2) identify variables associated with changed sports-related leisure behavior in the Alpine regions of Austria, Germany and Italy during the pandemic.

## 2. Materials and methods

### 2.1. Study design and data collection

The research topics addressed in this study were an optional part of a large-scale retrospective online survey examining changes in physical activity, health and lifestyle behaviors due to COVID-19 (15). Data collection was conducted from December 12, 2020, to January 31, 2021, after a pre-test period, using SoSci Survey online survey software (https://www.soscisurvey.de/). The survey was open to people aged 18 years and older who had at least a temporary residence in one of the five regions Tyrol (Austria), Vorarlberg (Austria), Upper Bavaria (Germany), South Tyrol (Italy) and Trentino (Italy) during the COVID-19 pandemic. The survey was available in German and Italian. At the beginning of the survey, respondents were informed about the purpose of the study, the length of the survey (which lasted on average 21 ± 8 min) and about privacy and data protection. The survey was approved by the Institutional Review Board of the Department of Sport Science (IRB) as well as the Board for Ethical Issues (BfEI) of the University of Innsbruck.

### 2.2. Measures

The first and compulsory part of the online questionnaire included socio-demographic data (e.g., gender, age, education), mental health status (K6 scale), lifestyle (e.g., leisure time, sleep duration, screen time) and physical activity (Eurobarometer 472 study) behaviors as well as questions about the COVID-19 pandemic (e.g., COVID-19 risk group, COVID-19 concerns, satisfaction with COVID-19 crisis management). For details on the measures of the first part of the questionnaire, please see Schöttl et al. (15).

Based on the physical activity behaviors analyzed in the first part of the survey, this study examines sports-related leisure behavior. The second and optional part of the survey included questions about leisure behavior related to sports events, travel intentions as well as new sports offerings. Four statements about whether sports events during the COVID-19 pandemic should be held given the current situation (answer 1: “All major sports events should be canceled or postponed,” answer 2: “Each sports event must be considered individually and decided on a case-by-case basis,” answer 3: “The majority of sports events should be held as scheduled, but behind closed doors and without spectators,” answer 4: “The majority of major sports events should be held as scheduled and in attendance of spectators.”) were followed by questions about the perceived importance of attending major sports events before and during the COVID-19 pandemic (“How important was attending a major sports event to you before the COVID-19 pandemic?” and “How important is attending a major sports event to you now?”) using a 4-point Likert scale (not at all important, not very important, rather important, very important). In addition, the perceived importance of media consumption of major sports events before and during the COVID-19 pandemic (“How important was watching a major sports event *via* media to you before the COVID-19 pandemic?” and “How important is watching a major sports event *via* media to you now?”) was asked using the same 4-point Likert scale. We also looked at travel restrictions because of COVID 19 measures (e.g., “Travel restrictions affected my holiday plans.”) on a 4-point Likert scale (strongly disagree, disagree, agree, strongly agree). If the question about the use of new sports offerings/formats during the COVID-19 measures was answered with “yes,” a free input field for individual entry of new sports offerings/formats was provided. In addition, participants were asked how their e-sports use (sports competitions on computer, console) had changed since the outbreak of the COVID-19 pandemic and the accompanying restrictions (much less, a little less, just as much, a little more, a lot more).

### 2.3. Data analysis

Characteristics of the sample relating to demographics and physical activities are reported in mean (M) and standard deviation (SD) for continuous variables and in number and percent for categorial variables. To show significant differences between the demographic variables and the three countries, a one-way ANOVA for continuous variables (age, BMI, household size) and an χ^2^-test for categorial variables were performed. Participants could choose one of four responses on how major sports events should be handled during the COVID-19 pandemic. To determine significant associations between the responses and categorial variables (country, gender, education, income, marital status, household composition, K6 scale, member of sports club, regular physical activity, COVID-19 positive, COVID-19 risk group), an χ^*2*^-test was conducted. For the association between the answers and continuous variables (household size, age), a one-way ANOVA and a Tukey *post-hoc* analysis for pairwise comparisons were used. To examine significant differences in vacation planning between individuals from Austria, Germany and Italy, a one-way ANOVA and a Tukey *post-hoc* analysis for pairwise comparisons were performed. If homogeneity of variance as verified with Levene's Test was not present in both previously mentioned analyses, the Welch-ANOVA and the Games-Howell *post-hoc* analysis were evaluated (33). To predict affected travel planning during COVID-19 due to travel restrictions with several predictor variables, a binary logistic regression was performed. The dependent variable (affected travel planning) was dichotomized by converting the 4-point Likert scale (strongly disagree, disagree, agree, strongly agree) into two outcomes: impact on travel planning (yes = agree) and no impact on travel planning (no = disagree). To identify a significant difference in the perceived importance of attending a major sports event and the perceived importance of watching a major sports event *via* media before the COVID-19 pandemic compared with the second lockdown in November/December 2020, paired *t*-tests (34) were conducted for participants from Austria, Germany and Italy. χ^2^-tests were used to determine significant associations between the variables “use of e-sports during COVID-19” and “use of new sports offerings/formats during COVID-19” and the variable “countries.” SPSS version 26 was used for all analyses and statistical significance was declared if *p* < 0.05.

## 3. Results

### 3.1. Participants

After data cleaning, *n* = 1,809 participants were included in the study. As the sample of the optional part was smaller than the first part of the questionnaire (*n* = 2,975), the five Alpine regions (Tyrol, Vorarlberg, Upper Bavaria, South Tyrol and Trentino) were grouped into three countries: Austria (Tyrol and Vorarlberg, *n* = 761), Germany (Upper Bavaria, *n* = 308) and Italy (South Tyrol and Trentino, *n* = 740). The gender distribution of the total sample was balanced, with 51.5% women and 48.4% men (0.2% other). The average age of the participants was 42.5 years, with a BMI (kg/m^2^) of 23.9 ± 3.8. 71.3% of respondents reported living in a partnership/marriage and only a few reported living alone (14.2%), with an average of 2.8 persons per household. The educational and income levels of the respondents were medium (48.5 and 33.5%, respectively) to high (43.3 and 41.0%, respectively) and the majority (93.5%) were not from a migrant background. The K6 scale showed that 11.9% of all respondents could be classified as probably having a severe mental illness (SMI). The majority did not belong to the COVID-19 risk group (91.1%). Regarding physical activity behavior, 59.6% of respondents stated they were a member of a sports club (18.3% fitness/health center) and 90.4% of participants reported that they practiced sports regularly (at least once a week). Most had access to a garden/terrace or balcony (94.0%). Table 1 provides detailed information on the sociodemographic and physical activity variables. There are significant differences between all variables despite marital status and countries. Due to the fact that the sociodemographic characteristics are very similar, countries can be compared.

**Table 1:** Demographic and physical activity-related characteristics of participants in the Alpine regions of Austria, Germany and Italy.

**Variable *N* (%) or mean ±SD**	**Austria (AUT) (*n* = 761)**	**Germany (GER) (*n* = 308)**	**Italy (ITA) (*n* = 740)**	**Total (*n* = 1,809)**	***p*-value**
**Gender**					< 0.001
Female	387 (50.9)	191 (62.0)	353 (47.7)	931 (51.5)	
Male	372 (48.9)	116 (37.7)	387 (52.3)	875 (48.4)	
Other	2 (0.3)	1 (0.3)	0 (0.0)	3 (0.2)	
**Age**	41.5 ± 14.0	41.8 ± 14.0	43.9 ± 13.5	42.5 ± 13.8	0.002^**a**^
**BMI**	24.4 ± 4.0	24.1 ± 4.1	23.3 ± 3.3	23.9 ± 3.8	< 0.001^**b**^
**Marital status**					0.197
Single	202 (26.5)	96 (31.2)	222 (30.0)	520 (28.7)	
Partner/married	559 (73.5)	212 (68.8)	518 (70.0)	2,109 (70.9)	
**Education**					< 0.001
Low	31 (4.1)	55 (17.9)	62 (8.4)	148 (8.2)	
Middle	404 (53.1)	99 (32.1)	374 (50.5)	877 (48.5)	
High	326 (42.8)	154 (50.0)	304 (41.1)	784 (43.3)	
**Personal monthly income (net)**					< 0.001
< € 1,000	107 (14.1)	52 (16.9)	140 (18.9)	299 (16.5)	
€1,000– < €2,000	205 (26.9)	69 (22.4)	332 (44.9)	606 (33.5)	
>€2,000	276 (49.4)	151 (49.0)	214 (28.9)	741 (41.0)	
No response	73 (9.6)	36 (11.7)	54 (7.3)	163 (9.0)	
**Household size**	2.8 ± 1.3	2.6 ± 1.2	3.1 ± 1.3	2.9 ± 1.3	< 0.001^**c**^
**Private access to garden/terrace/balcony**					0.035
No	43 (5.7)	28 (9.1)	37 (5.0)	108 (6.0)	
Yes	718 (94.3)	280 (90.9)	703 (95.0)	1,701 (94.0)	
**K6 Scale (*****N*** = **1,791)**					0.008
No SMI	684 (90.8)	263 (87.1)	631 (85.7%)	1,578 (88.1)	
SMI	69 (9.2)	39 (12.9)	105 (14.3)	213 (11.9)	
**COVID-19 risk group (*****N*** = **1,802)**					0.022
No	688 (90.6)	270 (87.7)	683 (92.9)	1,641 (91.1)	
Yes	71 (9.4)	38 (12.3)	52 (7.1)	161 (8.9)	
**Members of sports club (*****N*** = **1,787)**	446 (59.2)	158 (52.0)	461 (63.2)	1,065 (59.6)	0.004
**Members of fitness/health center (*****N*** = **1,787)**	150 (19.9	84 (27.6)	93 (12.7)	327 (18.3)	< 0.001
**Regular physical activity (at least once a week) (*****N*** = **1,799)**	708 (93.2)	276 (89.9)	642 (87.7)	1,626 (90.4)	0.002

χ^2^ tests and one-way ANOVA (age, BMI, household size) were conducted to evaluate the significance of differences in characteristics of three countries; *post-hoc* tests showed significant differences between the following countries:

a AUT and ITA,

b ITA and AUT, ITA and GER,

c AUT and GER, AUT and ITA, GER and ITA.

AUT, region of Tyrol and Vorarlberg; GER, region of Upper Bavaria, ITA, region of Trentino and South-Tyrol.

### 3.2. Handling of major sports events

The majority of respondents from the three countries (Austria: 57.2%, Germany: 50.8%, Italy: 53.4%) chose answer 2 (“Each sports event must be considered individually and decided on a case-by-case basis.”). As can be seen in Table 2 there is a significant relationship between six variables (countries, gender, household size, sports club members, K6 scale and regular sports participation) and the four response options on handling sports events. *Post-hoc* analysis reported a significant difference in household size of participants who chose answer 1 (“All major sports events should be canceled or postponed.”) and answer 4 (“The majority of major sports events should be held as scheduled and in attendance of spectators.”). There was no significant relationship between the variables age (*p* = 0.428), education (*p* = 0.094), income (*p* = 0.606), marital status (*p* = 0.516), household composition (*p* = 0.962), COVID-19 disease (*p* = 0.424), COVID-19 risk group (*p* = 0.231) and the response options.

**Table 2:** Handling of major sports events in the Alpine regions of Austria, Germany and Italy.

**Variable *N* (%) or mean ±SD**	**Answer 1**	**Answer 2**	**Answer 3**	**Answer 4**	***p*-value**
**Country**					< 0.001
Austria	113 (14.9)	433 (57.2)	156 (20.6)	55 (7.3)	
Germany	67 (21.8)	156 (50.8)	65 (21.2)	19 (6.2)	
Italy	91 (12.3)	394 (53.4)	216 (29.3)	37 (5.0)	
**Gender**					0.001
Female	154 (16.6)	528 (57.0)	197 (21.3)	47 (5.1)	
Male	116 (13.3)	454 (52.0)	239 (27.4)	64 (7.3)	
**Household size**	2.7 ± 1.2	2.8 ± 1.3	2.9 ± 1.3	3.2 ± 1.4	0.022
**K6 scale**					0.003
No SMI	235 (14.9)	869 (55.2)	384 (24.4)	85 (5.4)	
SMI	32 (15.2)	106 (50.2)	48 (22.7)	25 (11.8)	
**Members of sports club**					< 0.001
No	147 (20.5)	371 (51.7)	159 (22.1)	41 (5.7)	
Yes	121 (11.4)	600 (56.5)	272 (25.6)	69 (6.5)	
**Regular physical activity**					0.028
No	37 (21.4)	79 (45.7)	48 (27.7)	9 (5.2)	
Yes	232 (14.3)	897 (55.4)	388 (24.0)	102 (6.3)	

χ^2^ tests and one-way ANOVA (age, household size) were conducted to evaluate significant differences in variables and four response options (*n* = 1,802). Answer 1: “All major sports events should be canceled or postponed,” answer 2: “Each sports event must be considered individually and decided on a case-by-case basis,” answer 3: “The majority of sports events should be held as scheduled, but behind closed doors and without spectators,” answer 4: “The majority of major sports events should be held as scheduled and in attendance of spectators.”

### 3.3. Attendance and media consumption of major sports events

**Figure 1:**
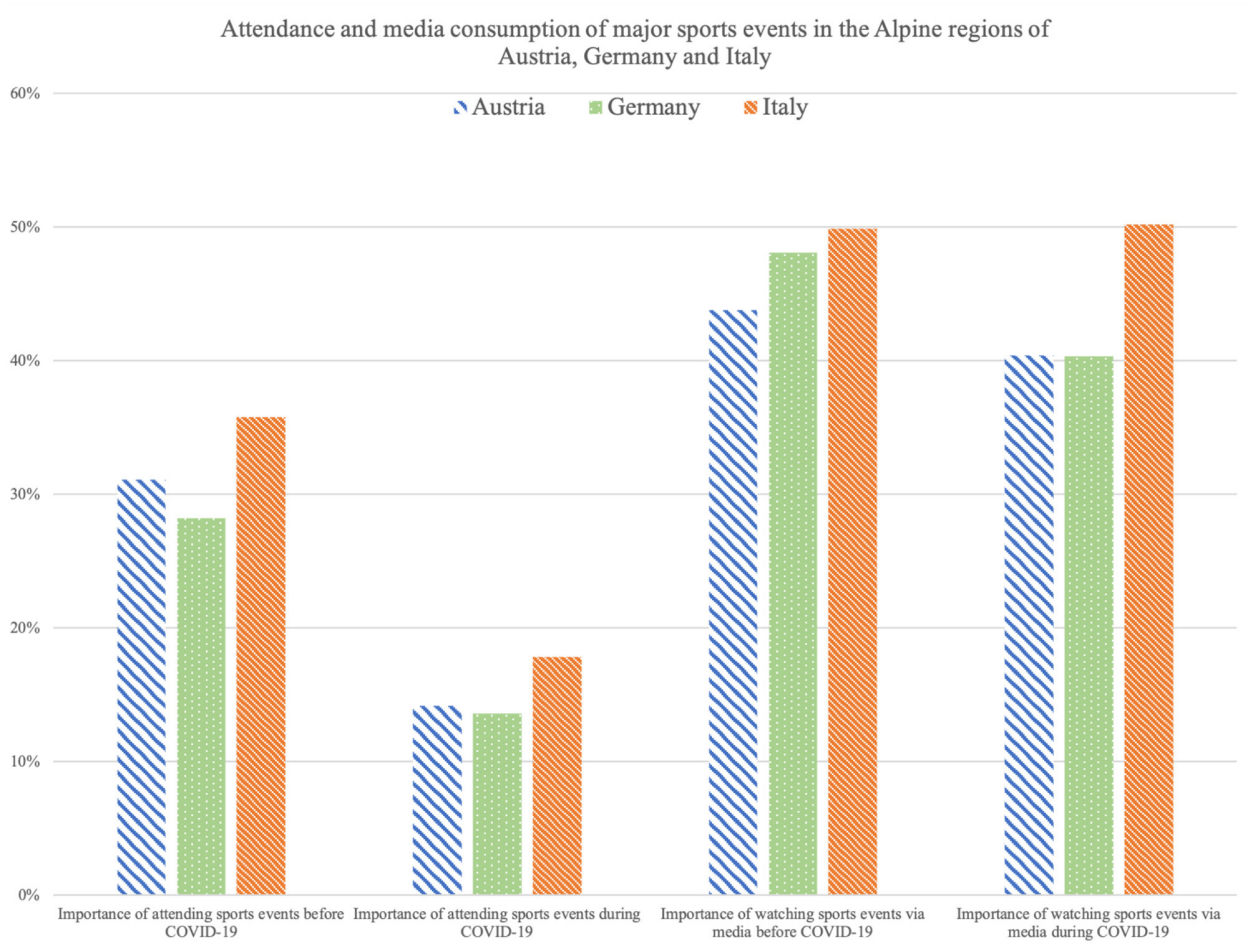
Self-reported importance of attending (onsite) and watching (*via* media) major sports events before and during COVID-19 in the Alpine regions of Austria, Germany, and Italy.

Participants from the three countries indicated the perceived importance they attached to watching major sports events onsite or *via* media over the two time periods before COVID-19 and during the second lockdown in November/December 2020. Figure 1 shows how important watching sports events, onsite and *via* media, was to participants from Austria, Germany and Italy before and during COVID-19, respectively. For simplified presentation, the 4-point Likert scale was summarized into “important” (rather important, very important) and “not important” (not very important, not at all important). The bar chart illustrates that the self-reported importance of attending major sports events decreased in all countries in the course of the COVID-19 pandemic (Austria: 31.1% before COVID-19 vs. 14.2% during COVID-19, Germany: 28.2% before COVID-19 vs. 13.6% during COVID-19, Italy: 35.8% before COVID-19 vs. 17.8% during COVID-19). A significant difference could be seen in the importance attached to attending major sports events before the COVID-19 pandemic compared with the second lockdown in November/December 2020 among participants from Austria (*p* < 0.001), Germany (*p* < 0.001) and Italy (*p* < 0.001). Similarly, the self-reported importance of watching major sports events *via* media decreased for participants in Germany (40.3%) compared to the time before the pandemic (48.1%). Participants from Austria recorded a slight decline (43.8 vs. 40.4%) and Italian participants showed an almost unchanged perceived importance of watching major sports events over these two points in time (49.9 vs. 50.2%). The self-reported importance of watching major sports events *via* media before the COVID-19 pandemic compared to the time during the pandemic declined significantly among participants in Germany (*p* < 0.001), while no significant differences were found between the two time points for participants in Austria (*p* = 0.217) and in Italy (*p* = 0.683).

### 3.4. Sports-related vacation planning during travel restrictions

Four participants' statements about sports-related vacation planning during COVID-19 travel restrictions were summarized as “disagree” (disagree, strongly disagree) and “agree” (agree, strongly agree) for the presentation of descriptive results. Over 80% of respondents (Austria: 84.5%, Germany: 85.9%, Italy: 80.4%) agreed that the travel restrictions had influenced their vacation planning. Significant differences (*p* < 0.001) between countries were shown between Italy and Austria and between Italy and Germany. From Austria, 62.7% of respondents stated that they wanted to spend their vacation in their own country due to travel restrictions. In Germany, the proportion was 57.1% and in Italy 75.1%. Significant differences (*p* < 0.001) were revealed between Italy and Austria and Italy and Germany, respectively. More than half of the respondents living in the Alpine regions of Austria (66.3%), Germany (65.5%) and Italy (56.9%) stated that sports offerings and opportunities played a major role when choosing a vacation destination. These differences were significant (*p* < 0.001) between Italy and Austria as well as between Italy and Germany. Approximately half of respondents indicated that travel restrictions had encouraged them to use domestic sports facilities (Austria: 48.6%, Germany: 51.0%, Italy: 49.1%). There is no significant difference between countries (*p* = 0.579).

A binary logistic regression examined associations between vacation planning during the COVID-19 pandemic and several variables. The binary logistic regression model was statistically significant [χ^*2*^ (14) = 47.32, *p* < 0.001], but resulted in a poorly explained variance verified by Nagelkerke's *R*^2^ (*R*^2^ = 0.048) (35). Goodness-of-fit was evaluated using the Hosmer-Lemeshow-Test, indicating a good model fit (*p* = 0.151). Nine variables were entered into the logistic regression model. Five variables (age, marital status, household composition, private access to garden/terrace/balcony, stress) did not contribute to predicting affected vacation planning during COVID-19 due to travel restrictions. Two variables and two categories of variables showed significant associations with affected vacation planning. Male respondents showed a lower probability that their vacation planning was influenced compared to females. Middle and higher incomes among the participants, compared to the reference category, increased the likelihood that vacation planning was influenced. Individuals whose quality of life and mental health worsened during COVID-19, relative to the reference category, were more likely to have had their vacation planning influenced. Please see Table 3 for detailed values of the logistic regression.

**Table 3:** Results of the binary logistic regression model examining associations between vacation planning during the COVID-19 pandemic and several variables (*n* = 1,809).

**Variable**	**Category**	**Affected vacation planning during COVID-19**		
		**Regression coefficient B**	**Odds ratio exp (B)**	**Sig**.
Gender	Female (Reference)	−0.427	0.652	**0.003**
	Male			
Age		−0.008	0.992	0.132
Income	Low (Reference)			
	Middle	0.454	1.574	**0.016**
	High	0.896	2.449	**< 0.001**
Marital status	Single (Reference)	0.043	1.043	0.815
	Partner/married			
Household composition	Living alone (Reference)			
	Living with others but no children	−0.126	0.881	0.603
	Living with children (including others)	−0.202	−0.202	0.430
Private access to garden/terrace/balcony	No (Reference)			
	Yes	0.356	1.428	0.195
Quality of life	Same during COVID-19 (Reference)			
	Decreased	0.316	1.372	**0.045**
	Increased	0.034	1.035	0.887
Mental health	Same during COVID-19 (Reference)			
	Decreased	0.360	1.433	**0.025**
	Increased	−0.192	0.825	0.466
Stress	Same during COVID-19 (Reference)			
	Less	−0.102	0.903	0.570
	More	0.002	1.002	0.989

The bold values indicate the statistically significant *p*-values (< 0.05).

### 3.5. Use of new sports offerings/formats and e-sports

Of all respondents, 31.9% (Austria: 32.5%, Germany: 40.3%, Italy: 27.8%) had used new sports offerings during the COVID-19 pandemic. χ^*2*^ showed a significant relationship between new sports offerings and the variable countries (*p* < 0.001). Participants specified the new sports offerings and formats they used during the COVID-19 pandemic in a free input field of the questionnaire. The entries were grouped into three categories: Online offerings, alternative/new sports offerings and home training. Online offerings (such as YouTube videos, social media content, fitness apps) were most frequently reported by about three quarters (72.4%), followed by alternative/new sports offerings accounting for 19.9% and home training for 7.7%. The use of e-sports changed during the COVID-19 pandemic. Approximately thirty percent of respondents (29.7%) increased their e-sports consumption, while some reported a decrease (6.8%) and 63.6% stated their e-sports consumption had remained the same. Table 4 shows e-sports usage by country. The χ^*2*^-test showed a significant correlation between the use of e-sports and the variable countries (*p* = 0.003).

**Table 4:** Use of e-sports during the COVID-19 pandemic in the Alpine regions of Austria, Germany and Italy.

**E-sport usage during COVID-19 *N* (%) (*n* = 590)**	**Austria**	**Germany**	**Italy**	**Total**
Less	19 (9.2)	9 (12.7)	12 (3.8)	40 (6.8)
The same	117 (56.8)	40 (56.3)	218 (69.6)	375 (63.6)
More	70 (34.0)	22 (31.0)	83 (26.5)	175 (29.7)

χ^*2*^-test showed a significant correlation between the use of e-sports and the variable countries (*p* = 0.003).

## 4. Discussion

In the present study, changes in sports-related leisure behavior were investigated in the Alpine regions of Austria, Germany and Italy. Behavioral changes were observed during the pandemic and compared to pre-COVID-19 pandemic levels in relation to handling, attending and media consumption of major sports events, travel and vacation behavior and the use of new sports offerings and formats. The results revealed that the implementation of major sports events needs to be considered individually and decided on a case-by-case basis. Furthermore, it was shown that the importance attached to watching sports events on-site decreased in the course of the COVID-19 pandemic, whereas following sports events *via* media declined in importance only for participants in Germany; for participants in Austria and Italy this level remained more or less constant. In the context of sports-related travel behavior, we demonstrated that travel restrictions had an impact on vacation planning, that holidays at home, including the use of local sports facilities and opportunities, were of increasing interest and that sports offerings in general played a major role in the choice of vacation destination. Physical activity behavior also showed a tendency toward the use of online services, such as fitness apps, YouTube videos or social media content. In this context, e-sports seemed to play an increasingly important role.

Attending sports events on-site became less important for the respondents of all three countries compared to media consumption during COVID-19. A potential fear of infection during crowd gatherings could be a possible reason for the change in the perceived importance of visiting major sports events. This consideration could also be related to the assumption that the pandemic raised intrapersonal constraints, such as the fear and anxiety of contracting the virus (3). As stadium visits for major sports events were not permitted for a certain period of time due to strict COVID-19 measures, different types of sports media broadcasts created opportunities for the production of new media consumption formats. A commentary from the US (36) examined how the sports industry developed media content strategies with the help of new, mixed and rebroadcasted content across multiple broadcast and streaming platforms to provide (new) media consumption possibilities. These initial ideas for a diverse broadcasting of live sports content represent a great opportunity, especially in times of pandemics and the associated risks, offering fans an entertaining live experience without having to visit sports facilities. As, at the time of writing, major sports events were open to spectators again, media strategies for the owners of content and rights could be enhanced by new ways of creating, combining and distributing content (36).

COVID-19 measures and travel restrictions affected holiday planning in the Alpine regions of the three countries. Our results showed that vacations within national boundaries became more important. The emerging trend toward domestic travel is consistent with the study by Kang et al. (3). This and other studies (37–39) showed that traveling close to home rather than to distant destinations and spending time in nature for leisure and recreation were important for vacation planning during geographical restrictions. The concept of “staycation” or vacationing in the local area established itself as a popular leisure time activity during travel restrictions and while borders were closed (40, 41). Moreover, staycations as a form of domestic tourism, which tend to be shorter in length and closer to home (compared to long-distance travel), are characterized by local activities and excursions, short (driving) distances and therefore more sustainable transport (5, 40, 42).

Of the three countries included in our study, respondents from the Alpine regions in Italy are most likely to spend their holidays in their own country. This could be explained by the fact that the strictest measures were implemented in Italy, that Italy had the longest closed period (10 weeks), and also that the respondents from Italy were on the lowest incomes compared to the other two countries. These structural constraints (3) on income, the inaccessibility of destinations due to closures and the difficulties in getting in touch with the relevant authorities (e.g., to obtain travel documents) were particularly evident in Italy.

In this context, the concept of “micro-escapes” also appeared to become more attractive, especially for people who were struggling financially due to the COVID-19 pandemic (3). Micro-escapes are short, affordable and convenient breaks and can include hiking, camping and weekend getaways (3, 43). Destination marketing organizations could pay attention to this concept and identify not only short-term travelers but also local residents as a target group during such a pandemic (3). Even after the opening of borders and the resumption of air travel, this trend could continue during an ongoing pandemic, not only for those facing structural constraints (e.g., income cuts) but also for those who fear infection (intrapersonal constraints). Consequently, low risk travel options under current conditions, such as staycations or micro-escapes in the near vicinity could pique the interest of individuals as long as these locations are in a relatively safe COVID-19 environment (5, 44). In this regard, tourism operators face the challenge of adapting staycation and micro-escape offerings to the changing preferences, motives and desires of tourists, taking into account changing psychological resources (5).

It should also be noted that travel can contribute to wellbeing (45, 46). Vacations have a positive effect on personal resources, contribute to relaxation, and can also help prevent serious health problems such as depression, stress, or heart disease (47, 48). These health benefits could be of particular relevance for future strategies to promote physical activity as well as in sports and health tourism.

Our results show that people from the Alpine regions of Austria, Germany and Italy took advantage of new exercise opportunities to stay physically fit during COVID-19. Around 32% of all respondents used new sports formats. Of those using new sports formats, around three-quarters took advantage of online offerings, such as web classes, health apps, or fitness videos. According to Nyenhuis et al. online home training on training apps, such as Mirror, Zwift, iFit and Nordic Track, is an emerging trend in the leisure context (49). Furthermore, Kaushal et al. found that most adults preferred to spend their leisure time participating in physical activities at home during the COVID-19 pandemic (6). These findings are consistent with our results, assuming that individuals participated in their online classes at home during COVID-19 restrictions. In addition, e-sports increased by around 30% across all three countries. Gyms and sports providers could take advantage of this trend to keep existing consumers and attract new customers with the help of state-of-the-art technologies. In addition, these physical activity operators could also offer personal online training or group sessions and provide their equipment, such as stationary bikes, with monitors that enable web-based live gaming and exercising with real-life and virtual opponents (3). The potential consequences of increasing online offers and e-sports usage should be explored in more detail in future in order to better understand the behavioral changes triggered by the pandemic.

Respondents from the Alpine regions of Germany showed the highest number of alternative and new sports activities compared to the other two countries. This may be due to the fact that restrictions in Germany were more relaxed compared to Austria and Italy. The Germans thus had better options to try alternatives and discover new sports offerings. This result shows participants' persistence in following their leisure activities and goes along with other findings indicating that people responded proactively to leisure constraints caused by COVID-19 measures (3). Based on this fact, policymakers could develop campaigns and programs to help people maintain their leisure and physical activity levels or try new sports-related indoor and outdoor leisure activities.

Despite its strengths, the present study also has some limitations. The sample included an over-representation of participants displaying high levels of physical activity, health and income. Although people in Alpine regions are characterized by higher participation in sports (9), the proportion of physically active, healthy and well-educated people in the general population of the three countries is lower (50–52). Furthermore, data was collected retrospectively in December 2020 and January 2021. Statements about sports-related leisure behavior before COVID-19 and during the second lockdown may therefore differ from the actual behavior during these periods. In this context, the potential for reporting bias arises when using self-reported questionnaires (53). Due to ethical guidelines, participants were able to leave parts of the questionnaire unanswered. However, this led to an incomplete data set, followed by data cleaning and data loss.

## 5. Conclusion

As spending time on leisure can increase life satisfaction (54), limitations to leisure activities induced by COVID 19 may have led to a decrease in quality of life (3). Therefore, we focused our study on how planetary health challenges, like the COVID-19 pandemic, affect the sports-related leisure behavior of individuals and population groups in the Alpine regions of Austria, Germany and Italy. With regard to the affected aspects of physical activity, we shed light on behavioral changes to derive initial socio-economic implications for policymakers and physical activity providers.

We were able to show that people's interest in attending major sports events onsite seemed to decline whereas media consumption of major sports events appeared to be constant, which could lead to new formats of live sports consumption. Moreover, the attractiveness of local sports offerings on home turf increased. As travel intentions shifted toward domestic tourism, new trends in the sports and health tourism industry might emerge. Moreover, prioritizing both visitors and locals could lead to the creation of added value in this sector in future. It is also apparent that people with an affinity for sport become more creative in looking for alternatives to maintain their level of physical activity. In this context, COVID-19 could have an accelerating effect, particularly with regard to e-sports, a trend that might be crucial in the near future, and especially for sports providers looking to come up with more differentiated sports offerings incorporating greater digitalization. To meet the diverse needs and differentiated demands of consumers now and in the future, socio-economic providers of physical activity of all kinds will likely need to rethink their supply from a multi-angle perspective. We therefore assume that strategic considerations for an achievable elastic supply of sports-related leisure opportunities require resilient organizational structures and a clearer understanding of current trends in leisure activities to support policy and decision makers, leading to further considerations on how to proceed with research in this multidimensional area of investigation.

To our knowledge, research on how individuals adapt their leisure behavior to constraints in times of a pandemic is scant. This study, however, sheds light on changes in sports-related leisure behavior in countries with similar characteristics. Finally, further studies, particularly longitudinal and cross-country surveys, are needed to gain further insights into the changing sports-related leisure behavior impacted by the COVID-19 pandemic.

## Data availability statement

The datasets presented in this article are not readily available. The data that supports the findings of this study are available from the authors upon reasonable request. Requests to access the datasets should be directed to A-MK, Anna-Maria.Kogler@uibk.ac.at.

## Ethics statement

The studies involving human participants were reviewed and approved by Institutional Review Board (IRB) of the Department of Sport Science as well as the Board for Ethical Issues (BfEI) of the University of Innsbruck. Written informed consent for participation was not required for this study in accordance with the national legislation and the institutional requirements.

## Author contributions

A-MK: article administration, supervision, visualization, conceptualization, formal analysis, and writing—original draft. A-MK and SS: methodology, funding acquisition, resources, validation, and writing—review and editing. SS: investigation and data analysis. All authors contributed to the article and approved the submitted version.
